# Oral Rosiglitazone Supplementation Mitigates Pulmonary Cellular Stress Response During Lipopolysaccharide‐Evoked Acute Lung Injury in Mice

**DOI:** 10.1155/carj/1600008

**Published:** 2026-08-05

**Authors:** Jun-Ping Wei, Wei Cao, Zhao-Fei Liu, Zhen Liu, Zhen-Zhen Cao, Xiu Lu, Jia-Le Li, Min-Min Tang, Dong-Xu Hua, Lin Fu, Jun Fei, Ling Zheng

**Affiliations:** ^1^ Department of Respiratory and Critical Care Medicine, Linquan County People’s Hospital, Fuyang 236499, Anhui, China; ^2^ Department of Respiratory and Critical Care Medicine, The Second Affiliated Hospital of Anhui Medical University, Hefei 230601, Anhui, China, ahmu.edu.cn; ^3^ Institute of Respiratory Diseases, The Second Affiliated Hospital of Anhui Medical University, Hefei 230601, Anhui, China, ahmu.edu.cn

**Keywords:** acute lung injury, inflammation, lipopolysaccharide, NF-κB p65, PPARγ, rosiglitazone

## Abstract

Cellular stress is implicated in the progression of lipopolysaccharide (LPS)‐evoked acute lung injury (ALI). The goal of this study was to evaluate the effect of oral supplementation with rosiglitazone (RSG), which is an agonist of peroxisome proliferator‐activated receptor (PPAR)‐γ, on pulmonary inflammatory response, oxidative stress, and endoplasmic reticulum (ER) stress during LPS‐evoked ALI in mice. The mice were randomly divided into four groups and orally supplemented with or without RSG (10 mg/kg) once daily for 5 consecutive days before LPS (1 mg/kg) intraperitoneal injection. The mice were sacrificed at 12 h after LPS injection. As expected, LPS‐evoked ALI and death were attenuated in mice that were pretreated with RSG. LPS‐evoked increase in chemokines (KC, MIP‐2, and MCP‐1) and proinflammatory cytokines (TNF‐α and IL‐1β) was inhibited in the mouse lungs by RSG. RSG pretreatment significantly mitigated LPS‐evoked nuclear translocation of NF‐κB p65 and p50, which regulate inflammatory gene transcription in the lungs. Additionally, LPS‐evoked glutathione depletion and lipid peroxidation were alleviated by RSG. The abnormal expression of NADPH oxidases and antioxidant enzymes was restored in the lung tissues by RSG. Further experiments revealed that LPS‐evoked upregulation of GRP78, *p*‐IRE1α, and *p*‐EIF2α was blocked by RSG pretreatment. Mechanistically, RSG pretreatment enhanced PPARγ activity in the mouse lungs and bronchial epithelial cells. RSG promoted the interaction between NF‐κB p65 and PPARγ in the mouse lungs. Overall, RSG pretreatment protects against LPS‐evoked ALI partially through enhancing PPARγ activity and inhibiting the cellular stress response in the mouse lungs.

## 1. Introduction

Acute lung injury (ALI) and its more serious form, acute respiratory distress syndrome (ARDS), are universally life‐threatening diseases, which has a high incidence rate and mortality all over the world [1]. ALI/ARDS mainly manifests as a decline in lung compliance, noncardiogenic alveolar edema, progressive hypoxemic respiratory failure, and nonhydrostatic bilateral lung infiltration [2, 3]. Epidemiological research indicated that the incidence rates of ALI and ARDS are 78.9 and 58.7 per 100,000 patients in America, respectively. The total mortality of ARDS is almost 40% [4]. In addition, the morbidity of ARDS patients in intensive care unit (ICU), whose age is more than 15 years, is 2%, and the death rate is 70% in Shanghai, China [5]. More seriously, the morbidity and mortality of ALI are rising with age [6]. The pathogenesis of ALI/ARDS involved the disturbances in inflammation/anti‐inflammation, oxidants/antioxidants, and endoplasmic reticulum (ER) stress, ultimately resulting in lung injury [7]. Therefore, therapeutic measures of ALI/ARDS are targeted to suppress lung inflammation, which helps to alleviate clinical symptoms and increases pulmonary function [8].

Lipopolysaccharide (LPS), which is generally present in the digestive tracts of humans, is a toxic substance of cytoderm in Gram‐negative bacteria [9]. Increasing studies have demonstrated that LPS exposure induces ALI in mice [10, 11]. LPS exposure can directly lead to inflammation in pulmonary epithelial cells [12]. Our recent study suggested that LPS exposure could incur ALI and pulmonary inflammation in mice [13]. Peroxisome proliferator‐activated receptor (PPAR)‐γ is a member of the nuclear receptor family of ligand‐activated transcription factors with immunomodulatory properties which regulates lipid and glucose homeostasis, and has anti‐inflammatory and antioxidant activity [14]. Previous research has hinted that rosiglitazone (RSG), which is an agonist of PPARγ, can alleviate acute liver injury and inflammatory signaling activation [15]. Moreover, PPARγ activation inhibits the pathophysiology process of lung cancer, lung fibrosis, and chronic obstructive pulmonary disease (COPD) [16–18]. Nevertheless, the influences of RSG treatment on LPS‐evoked ALI and the potential mechanisms have not been clearly elucidated.

In the present study, the aim was to estimate the influence of RSG treatment on LPS‐evoked ALI in mice. Moreover, to better understand the mechanisms of RSG‐inhibited LPS‐evoked ALI, the levels of inflammatory cytokines and several signaling pathways associated with inflammation, oxidative stress, and ER stress were detected in the lungs of ALI models. The potential mechanisms were explored in human bronchus epithelial cells. Our results showed that oral RSG supplementation could mitigate ALI and pulmonary inflammation induced by LPS. Our results provide new evidence for the function of PPARγ as a significant therapeutic drug for LPS‐evoked ALI.

## 2. Materials and Methods

### 2.1. Chemicals and Reagents

LPS (*Escherichia coli* LPS, serotype 0127: B8) and RSG were bought from Sigma Chemical Company (St. Louis, MO). Primary antibodies against nuclear factor‐kappa B p65 (NF‐κB p65), NF‐κB p50, and β‐actin were obtained from Cell Signaling Technology (Beverley, MA). NOX‐2, NOX‐4, HO‐1, GRP78, *p*‐IRE1α, *p*‐EIF2α, and PPARγ antibodies were from Abcam (Cambridge, MA). Chemiluminescence (ECL) detection kits were purchased from Advansta Company. TRIzol reagent was purchased from Molecular Research Center, Inc. (Cincinnati, Ohio). RNase‐free DNase was purchased from Promega Corporation (Madison, WI). BCA protein assay kits were from Beyotime (China). All other reagents were from Sigma or as indicated in the methods.

### 2.2. Animals and Treatments

Eight‐week‐old Balb/c mice (male, 22–24 g) were purchased from Beijing Vital River Laboratory Animal Technology Co Ltd. The mice were given free food and free access to water at all times. The mice were adaptively fed for 5 days. These investigations included two independent animal experiments. In experiment 1, to evaluate the influence of oral RSG supplementation on LPS‐evoked ALI in mice, 48 mice were randomly divided into four groups: control group, RSG group, LPS group, and RSG + LPS group. In the RSG and RSG + LPS groups, the mice were orally supplemented with RSG (10 mg/kg) once daily for 5 consecutive days [19]. In the LPS and RSG + LPS groups, the mice were intraperitoneally injected with LPS (1 mg/kg) at 2 h after the last RSG treatment [13]. The mice were sacrificed at 12 h after LPS exposure. Lung specimens and serum samples were collected. In experiment 2, to assess the effect of RSG pretreatment on LPS‐evoked death, 48 mice were randomly divided into four groups: control group, RSG group, LPS group, and RSG + LPS group. The methods of RSG and LPS treatment were similar to those in experiment 1. Mice death was observed until 72 h after LPS injection.

### 2.3. Western Blotting

Lung tissues were collected, and total lung proteins were prepared using ice‐cold lysis buffer. The concentration of total protein was detected using a bicinchoninic acid (BCA) protein assay kit. The experimental steps were described in our previous study [20]. Briefly, the protein lysates were separated via 10% sodium dodecyl sulfate‐polyacrylamide gel electrophoresis, and transferred to polyvinylidene difluoride (PVDF) membranes. The membranes containing the target proteins were blocked in skim milk for 1.5 h at room temperature. Then, the membranes were incubated with primary antibodies and corresponding secondary antibodies for different times at 25°C. The blots were washed and then visualized with an enhanced chemiluminescence (ECL) reagent. The corresponding intensities of the protein bands were calculated after normalization to β‐actin using Image‐Pro Plus software.

### 2.4. Reverse Transcription‐Polymerase Chain Reaction (RT‐PCR)

The experimented RT‐PCR procedure was performed as previously depicted with minor modifications [21]. Total RNA was extracted from lung tissue via TRIzol reagent kits. The level of RNA was detected at wavelengths of 260 nm and 280 nm. mRNA was reverse transcribed to cDNA using avian myeloid leukemia virus reverse transcriptase kit (Promega). RT‐PCR was performed with a Light Cycler 480 SYBR Green I kit (Roche). The gene sequences are listed in Table 1. Target gene expression was analyzed and presented as the ratio to the reference gene (18S).

**TABLE 1:** Oligonucleotide sequences and size of primers.

Genes	Sequences	Sizes (bp)	Species
18S	Forward: 5′‐ GTAACCCGTTGAACCCCATT‐3′	151	Mouse
	Reverse: 5′‐ CCATCCAATCGGTAGTAGCG‐3′		

P47phox	Forward: 5′‐ TGGAGGGCAGAGACAATCCA‐3′	121	Mouse
	Reverse: 5′‐ GACGTCAGCTTCCGTTTGGT‐3′		

P67phox	Forward: 5′‐ TTCCCCTTGCAAGACTTACCC ‐3′	153	Mouse
	Reverse: 5′‐ TCCCCCTCCTCCATCTGTAAG ‐3′		

P22phox	Forward: 5′‐ TGGTATTTCGGCGCCTACTC ‐3′	106	Mouse
	Reverse: 5′‐ CCGACAACCATCGCTCCAT ‐3′		

Sod‐1	Forward: 5′‐ GGAAGCATGGCGATGAAAGC ‐3′	141	Mouse
	Reverse: 5′‐ CCCATGCTGGCCTTCAGTTA ‐3′		

Sod‐2	Forward: 5′‐ CTGTCCGATGATGTCAGCCA ‐3′	126	Mouse
	Reverse: 5′‐ TGCCGCTACTGAGAAAGGTG ‐3′		

Sod‐3	Forward: 5′‐ GGCCTTCTTGTTCTACGGCT ‐3’	84	Mouse
	Reverse: 5′‐ GCTAGGTCGAAGCTGGACTC ‐′		

Tnf‐α	Forward: 5′‐ ACCCTCACACTCACAAACCA ‐3′	134	Mouse
	Reverse: 5′‐ ACAAGGTACAACCCATCGGC ‐3′		

Il‐1β	Forward: 5′‐ TGCCACCTTTTGACAGTGATG ‐3′	138	Mouse
	Reverse: 5′‐ TGATGTGCTGCTGCGAGATT ‐3′		

Tnf‐β	Forward: 5′‐ TCACCTCAGACAGGACCCAT ‐3′	106	Mouse
	Reverse: 5′‐ AGCAGTGGCTGGCTTTTAGA ‐3′		

Kc	Forward: 5′‐ACTCAAGAATGGTCGCGAGG ‐3′	123	Mouse
	Reverse: 5′‐ GTGCCATCAGAGCAGTCTGT ‐3′		

Mip‐2	Forward: 5′‐ CAAAGGCAAGGCTAACTGACC ‐3′	123	Mouse
	Reverse: 5′‐ GAGGCACATCAGGTACGATCC ‐3′		

Mcp‐1	Forward: 5′‐ TAAAAACCTGGATCGGAACCAAA ‐3′	120	Mouse
	Reverse: 5′‐ GCATTAGCTTCAGATTTACGGGT ‐3′		

Grp78	Forward: 5’‐ CTGGCCGAGACAACACTGACCT ‐3′	68	Mouse
	Reverse: 5′‐ GCGACGACGGTTCTGGTCTCAC‐ 3′		

### 2.5. Lung Histology and Immunohistochemistry (IHC)

Left middle lung specimens were collected, fixed in 4% formalin and embedded in paraffin according to the previous study [22]. Paraffin‐embedded lung tissues were serially sectioned and subjected to conventional dewaxing. Then, the sections (5 mm) were stained with hematoxylin and eosin (H&E). The pathomorphology of the mouse lungs was observed by H&E staining under a light microscope. Then, the levels of NF‐κB p65, NF‐κB p50, PPARγ, and GRP78 were measured by IHC. Briefly, endogenous peroxidases were quenched with 3% H_2_O_2_. The antigen was repaired, and nonspecific binding was removed with 1% serum in PBS. After being blocked, the sections were incubated with primary antibodies and different secondary antibodies at room temperature. Afterward, the nuclei were counterstained with diaminobenzidine (DAB). The coverslips were placed on glass microscope slides and fixed using a neutral resin [23, 24]. The number of positive nuclei was estimated by two independent researchers who had no previous knowledge of the experimental design.

### 2.6. Cell Culture and Treatments

The human bronchus epithelial cell (BEAS‐2B) line was purchased from the American Type Culture Collection (USA). BEAS‐2B cells were cultured in RPMI‐1640 medium supplemented with fetal bovine serum (FBS). The cells were grown in a humidified incubator at 37 °C with 5% CO_2_. Moreover, the culture medium was replaced every 2 days. In order to explore the mechanism by which RSG pretreatment inhibited LPS‐evoked inflammation, BEAS‐2B cells were seeded into 75‐cm^2^ flasks. When the cell density reached 50%, BEAS‐2B cells were pretreated with RSG (10^−6^ M) at 24 h and 12 h before LPS (2 μg/mL) exposure [25]. At 24 h after LPS incubation, the cells were harvested and the relative experiments were conducted.

### 2.7. Coimmunoprecipitation (Co‐IP)

The homogenates of BEAS‐2B cells were prepared in lysis buffer (0.6% Nonidet P‐40, 150 mM NaCl, 5 mM EDTA, 1% Triton X‐100, 10% glycerin) containing 1% protease inhibitors [26]. Nuclear proteins were extracted. Cell homogenates (500 μg) were suctioned and precleared with protein A/G‐agarose (Santa Cruz). Next, PPARγ antibody was added and incubated at 4°C overnight. The agarose magnetic beads were washed using cold nondenaturing lysis buffer. Then, the agarose magnetic beads were centrifuged and gathered. The proteins were boiled, and the expression levels of NF‐κB p65 and PPARγ were detected through western blotting. The interaction between PPAR*γ* and NF‐κB p65 was analyzed.

### 2.8. Immunofluorescence (IF)

BEAS‐2B cells were seeded into 24‐well plates. The cell climbing films were pretreated with polylysine. BEAS‐2B cells were treated with RSG and LPS as previously described. Then, the cells were fixed with paraformaldehyde and blocked with serum at room temperature for different times. Next, the cell climbing films were washed with PBS and incubated with primary antibodies (PPARγ and NF‐κB p65) at 4°C in a humidified box. Then, the primary antibodies were collected and the corresponding fluorescent secondary antibodies were added and incubated in a darkroom. Finally, the nuclei were stained using 4’,6‐diamidino‐2‐phenylindole (DAPI). The localization of PPARγ and NF‐κB p65 was observed under a fluorescence microscope (Zeiss, Germany) [27].

### 2.9. Pulmonary GSH and MDA Detection

Lung tissues were collected, and homogenates were prepared. Commercial glutathione (GSH) assay kits were bought from Beyotime Biotechnology, China. Malondialdehyde (MDA) kits were obtained from Nanjing Jiancheng Bioengineering Institute, China. The contents of GSH and MDA were evaluated in the lungs according to the manufacturer’s instructions [28].

### 2.10. Enzyme‐Linked Immunosorbent Assay (ELISA)

The blood specimens were collected from the different groups of mice. Following immobilization for 1.5 h at room temperature and centrifugation at 4000 g for 10 min, serum samples were obtained and stored in an ultralow‐temperature refrigerator. The levels of serum tumor necrosis factor (TNF)‐α and keratinocyte chemoattractant (KC) were detected using ELISA. TNF‐α (CSB‐E04741m) and KC (CSB‐E17286m) ELISA kits were obtained from Cusabio (Wuhan, China) (https://www.cusabio.com/). Based on the manufacturer’s instructions, 50 μL of the standards or test samples were added to the well, and then the microplate was incubated for 2 h at 37°C. After washing with washing buffer, 100 μL of biotin‐labeled antibody was added, and subsequently hatched with 100 μL of HRP‐streptavidin conjugate. Afterward, 90 μL of TMB substrate was added for 0.5 h at 37°C. At 30 min after the addition of the stop solution, the optical density was read at a wavelength of 450 nm with a microplate reader. The concentrations of serum inflammatory cytokines were calculated according to the standard curve.

### 2.11. Statistical Analysis

All statistical analyses were conducted using Statistical Package of Social Sciences software (SPSS) version 18.0. One‐way ANOVA and Student–Newman–Keuls post hoc tests were used to assess the differences in variables among different groups. The differences of variables between two groups were compared with Student’s *t*‐test. Statistical significance was defined at *p* < 0.05.

## 3. Results

### 3.1. Oral RSG Supplementation Relieved LPS‐Evoked ALI in Mice

**FIGURE 1:**
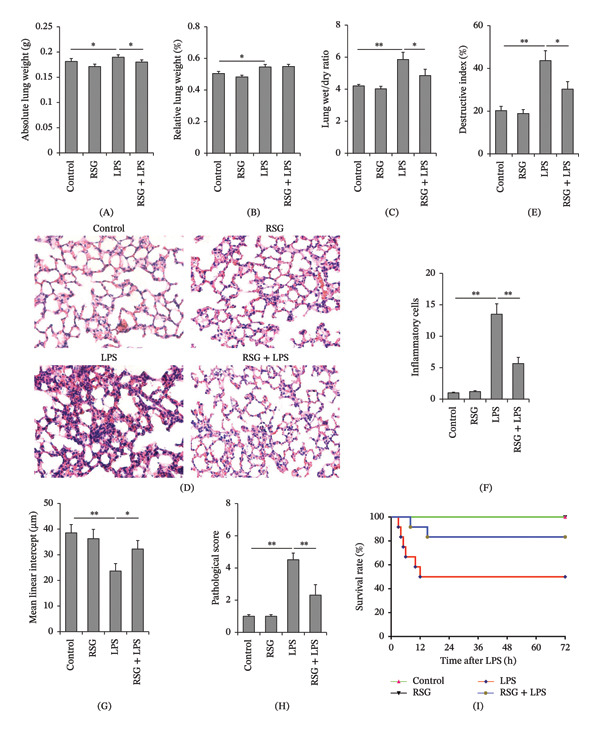
The effect of oral RSG supplementation on LPS‐evoked ALI in mice. The male mice were randomly divided into four groups. In the RSG and RSG + LPS groups, the mice were orally supplemented with RSG (10 mg/kg) once daily for 5 consecutive days. In the LPS and RSG + LPS groups, the mice were intraperitoneally injected with LPS (1 mg/kg) at 2 h after the last RSG treatment. The mice were sacrificed at 12 h after LPS exposure. Lung tissues and serum samples were collected. (A) Absolute lung weight; (B) relative lung weight; (C) lung wet/dry ratio; (D) pulmonary histopathology; (E) destructive index; (F) inflammatory cells; (G) mean linear intercept; (H) pathological score; (I) survival rate. Data were expressed as mean ± S.E.M. (*N* = 12). ^∗^ *p* < 0.05, ^∗∗^ *p* < 0.01.

The effect of oral RSG supplementation on LPS‐evoked ALI was estimated in mice. As shown in Figure 1A–C, the absolute lung weight, relative lung weight, and lung wet/dry ratio were obviously elevated in 12 h after LPS injection. There was no influence of oral RSG supplementation on the LPS‐induced increase in relative lung weight. However, pretreatment with RSG obviously attenuated the LPS‐evoked increase in absolute lung weight and in lung wet/dry ratio of mice (Figure 1A–C). Moreover, the effect of RSG pretreatment on pulmonary pathology was analyzed. As shown in Figure 1D, alveolar structure was injured, and significant pulmonary edema and inflammatory cell infiltration were observed in the lungs of LPS‐injected mice. In addition, LPS exposure remarkably elevated the destructive index of the alveolar structure and inflammatory cells, and downregulated the mean linear intercept in the lungs (Figure 1E–G). The pathological score was substantially upregulated in the lungs of LPS‐injected mice (Figure 1H). As expected, oral RSG supplementation dramatically reversed LPS‐evoked pulmonary damage and pathological changes (Figure 1E–H). Besides, supplementation with RSG significantly mitigated LPS‐evoked mice death within 72 h of LPS injection (Figure 1I).

### 3.2. Oral RSG Supplementation Mitigated LPS‐Activated Pulmonary NF‐κB Signaling in Mice

**FIGURE 2:**
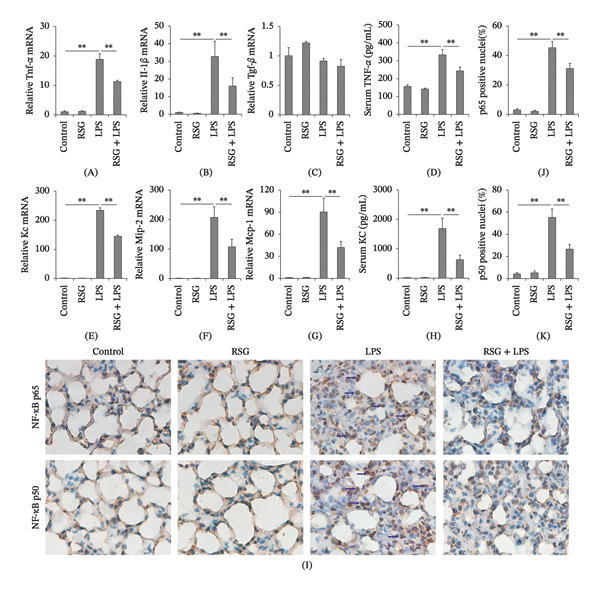
The effect of oral RSG supplementation on LPS‐activated NF‐κB signaling in the mouse lungs. The male mice were randomly divided into four groups. The mice were administered with RSG or LPS as described in the Materials and Methods section. Lung tissues and serum samples were collected. The levels of proinflammatory cytokines and chemokines were detected in the lungs and serum. (A–C) The levels of proinflammatory cytokine genes were detected in the lungs through RT‐PCR. (A) Tnf‐α; (A) Il‐1β; (C) Tgf‐β. (E–G) The expressions of chemokines genes were measured in the lungs via RT‐PCR. (E) Kc; (F) Mip‐2; (G) Mcp‐1. (D, H) The levels of proinflammatory cytokines and chemokines were evaluated by ELISA. (D) TNF‐α; (H) KC. (I–K) The numbers of NF‐κB p65‐and NF‐κB p50‐positive nuclei were observed through IHC. (I) Representative pictures of NF‐κB p65‐and NF‐κB p50‐positive nuclei were shown. (J) Quantitative analysis of NF‐κB p65‐positive nuclei. (K) Quantitative analysis of NF‐κB p50‐positive nuclei. Data were expressed as mean ± S.E.M. (*N* = 6). ^∗∗^ *p* < 0.01.

The effect of oral RSG supplementation on LPS‐evoked inflammatory cell secretion was estimated in the lungs of mice. As shown in Figure 2A–C, the level of transforming growth factor‐β (Tgf‐β) mRNA was not changed. The mRNA expressions of pulmonary Tnf‐α and interleukin‐1β (Il‐1β), which are two proinflammatory genes, were obviously increased in the lungs of LPS‐exposed mice. Serum TNF‐α expression was dramatically elevated at 12 h after LPS injection (Figure 2D). Of interest, RSG pretreatment obviously alleviated LPS‐evoked upregulation of Tnf‐α and Il*-*1β in the lungs (Figure 2A–D). Moreover, the expressions of Kc, macrophage inflammatory protein 2 (Mip‐2), and macrophage chemoattractant protein‐1 (Mcp‐1), three chemokine genes, were elevated in the lungs after LPS exposure. Furthermore, the level of serum KC was increased in the LPS‐injected mice. Interestingly, oral RSG supplementation obviously mitigated LPS‐evoked upregulation of chemokines in mice (Figure 2E–H). Then, NF‐κB signaling was detected in the lungs of mice. As shown in Figure 2I–K, NF‐κB p65 and NF‐κB p50, which are two subunits of NF‐κB, were activated in the lungs of LPS‐exposed mice at 12 h. Moreover, oral RSG supplementation notably inhibited LPS‐mediated nuclear translation of NF‐κB p65 and NF‐κB p50 in the lungs (Figure 2I–K).

### 3.3. Oral RSG Supplementation Alleviated LPS‐Evoked Pulmonary Oxidative Stress in Mice

**FIGURE 3:**
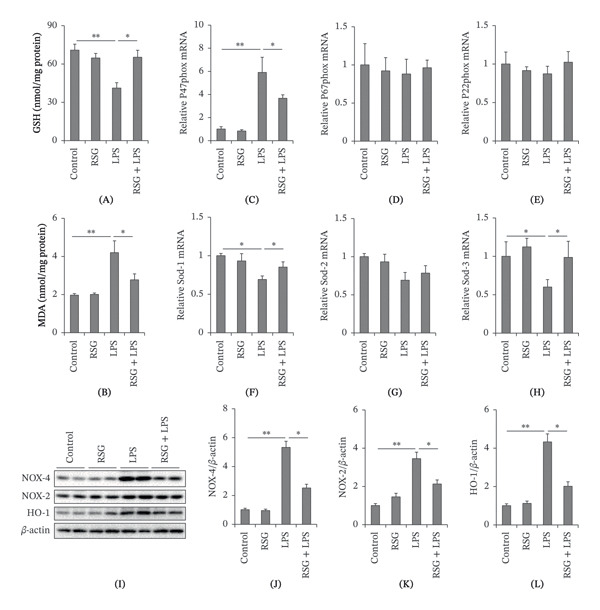
The effect of oral RSG supplementation on LPS‐evoked oxidative stress in the mouse lungs. All male mice were randomly divided into four groups. The mice were administered with RSG or LPS as described in the Materials and Methods section. Lung tissues and serum samples were collected. The markers of oxidative stress were detected. (A) The level of GSH was detected in the lungs. (B) The level of MDA was measured in the lungs. (C–E) The expressions of NADPH oxidase genes were analyzed in the lungs through RT‐PCR. (C) P47phox; (D) P67phox; (E) P22phox. (F–H) The levels of antioxidant enzyme genes were assessed in the lungs using RT‐PCR. (F) Sod‐1; (G) Sod‐2; (H) Sod‐3. (I–L) The protein expressions of NADPH oxidase and antioxidant enzyme were detected in the lungs by western blotting. (I) Representative bands. Quantitative analysis of scanning densitometry was conducted. (J) NOX‐4; (K) NOX‐2; (L) HO‐1. Data were expressed as mean ± S.E.M. (*N* = 6). ^∗^ *p* < 0.05, ^∗∗^ *p* < 0.01.

The effects of oral RSG supplementation on LPS‐evoked pulmonary GSH depletion and lipid peroxidation were assessed. Pulmonary GSH levels were significantly reduced, and pulmonary MDA, a marker of lipid peroxidation, was obviously elevated at 12 h after LPS exposure. In contrast, LPS‐evoked GSH depletion and lipid peroxidation were reversed by RSG pretreatment (Figure 3A,B). Moreover, pulmonary P47phox mRNA, a dinucleotide phosphate (NADPH) oxidase subunit, was dramatically elevated after LPS injection; oral RSG supplementation attenuated LPS‐evoked upregulation of pulmonary P47phox mRNA (Figure 3C). However, there was no obvious influence of LPS exposure on the levels of P67phox and P22phox in mice (Figure 3D, E). In addition, pulmonary superoxide dismutase (Sod) 1 and Sod3, two antioxidant enzyme genes, were prominently declined after LPS injection; however, LPS had no obvious effect on the level of Sod2 mRNA (Figure 3F–H). Interestingly, the LPS‐evoked decrease of pulmonary Sod1 and Sod3 was markedly mitigated in RSG‐pretreated mice (Figure 3F–H). Moreover, the LPS‐evoked upregulation of NADPH oxidase (NOX)‐2 and NOX‐4 was substantially restrained by oral RSG supplementation (Figure 3I–K). In addition, the expression of heme oxygenase‐1 (HO‐1), an inducible enzyme associated with oxidative stress, was obviously increased in the lungs after LPS injection. In contrast, supplementation with RSG suppressed the LPS‐induced upregulation of HO‐1 expression in the lungs (Figure 3I, L).

### 3.4. Oral RSG Supplementation Attenuated LPS‐Evoked Pulmonary ER Stress in Mice

**FIGURE 4:**
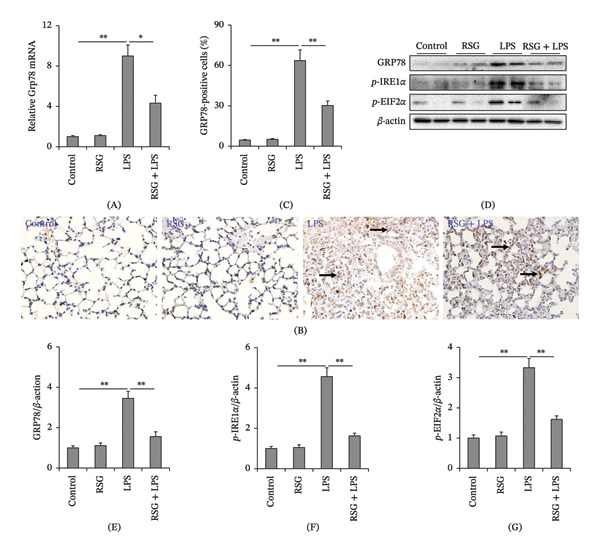
The effect of oral RSG supplementation on LPS‐evoked ER stress in the mouse lungs. All male mice were randomly divided into four groups. The mice were administered with RSG or LPS as described in the Materials and Methods section. Lung tissues and serum samples were collected. (A) The level of Grp78 mRNA was determined in the lungs by RT‐PCR. (B and C) The protein localization of pulmonary GRP78 was detected through IHC. (B) Representative pictures of GRP78‐positive cells. (C) Quantitative analysis of GRP78‐positive cells. The pulmonary markers of ER stress were measured with western blotting. (D) Representative bands. Quantitative analysis of scanning densitometry was conducted. (E) GRP78; (F) *p*‐IRE1α; (G) *p*‐EIF2α. Data were expressed as mean ± S.E.M. (*N* = 6). ^∗^ *p* < 0.05, ^∗∗^ *p* < 0.01.

The effect of RSG supplementation on LPS‐evoked pulmonary ER stress was evaluated during LPS‐evoked ALI. As shown in Figure 4A, the level of Grp78 mRNA was obviously increased in LPS‐exposed mice. Interestingly, LPS‐evoked Grp78 elevation was inhibited by RSG pretreatment in the lungs (Figure 4A). Moreover, LPS‐evoked upregulation of GRP78‐positive cells and protein expression in the lungs were obviously alleviated by RSG pretreatment (Figure 4B–E). In addition, the expressions of pulmonary *p*‐IRE1α and *p*‐EIF2α were obviously elevated at 12 h after LPS injection. As expected, oral RSG supplementation notably mitigated LPS‐triggered phosphorylation in IRE1α and EIF2α (Figure 4D,F,G).

### 3.5. Oral RSG Supplementation Activated Pulmonary PPAR*γ* Signaling in Mice

**FIGURE 5:**
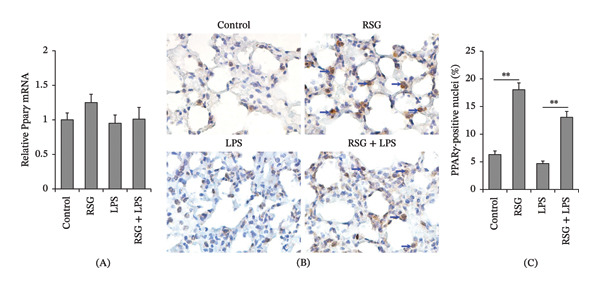
The effect of oral RSG supplementation on pulmonary PPARγ signaling in the mouse lungs. The mice were randomly divided into four groups. The mice were administered with RSG or LPS as described in the Materials and Methods section. Lung specimens and serum samples were collected. (A) The expression of PPARγ mRNA was detected using RT‐PCR. Nuclear PPARγ was determined in mouse lungs through IHC. (B) Representative pictures of PPARγ‐positive nuclei were presented. (C) Quantitative analysis of PPARγ‐positive nuclei was conducted. Data were expressed as mean ± S.E.M. (*N* = 6). ^∗∗^ *p* < 0.01.

The effect of oral RSG supplementation on pulmonary PPARγ activity was estimated. Although RSG pretreatment had no obvious influence on pulmonary Pparγ mRNA level (Figure 5A), the number of PPARγ‐positive nuclei was significantly upregulated in the lungs of RSG‐pretreated mice (Figure 5B, C). Moreover, there was no notable effect of LPS exposure on pulmonary PPARγ‐positive nuclei in mice (Figure 5B, C).

### 3.6. RSG Pretreatment Promoted the Interaction Between PPAR*γ* and NF‐κB p65 in Bronchial Epithelial Cells and Mouse Lungs

**FIGURE 6:**
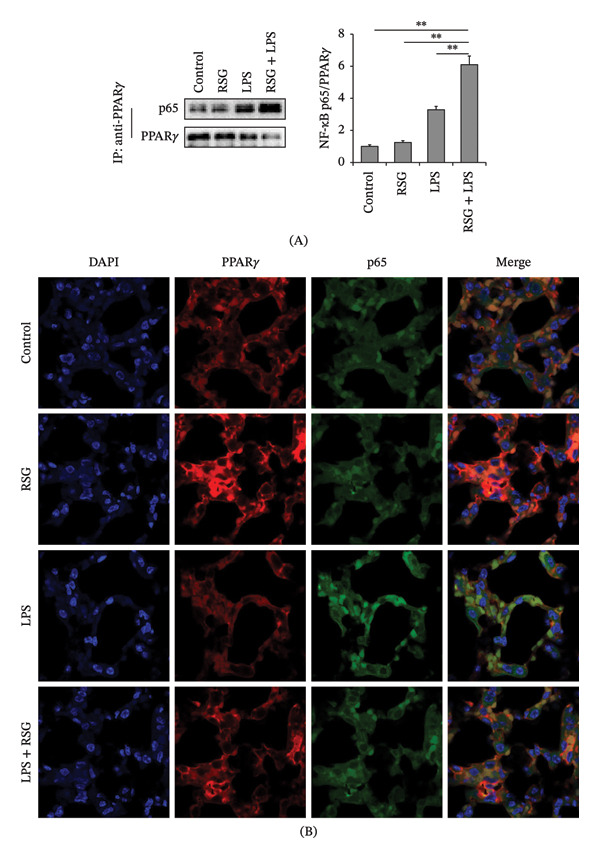
The interaction between PPARγ and NF‐κB p65 in BEAS‐2B cells and mouse lungs. (A) BEAS‐2B cells were incubated with RSG (10^−6^ (M) twice at 24 h and 12 h before LPS (2 μg/mL) exposure. After 24 h of LPS incubation, the cells were harvested. Nuclear protein was isolated. PPARγ was precipitated in BEAS‐2B cells. The expressions of PPARγ and NF‐κB p65 were detected using western blotting. (A) Representative bands of PPARγ and NF‐κB p65, and quantitative analysis of the interaction was performed. (B) The mice were randomly divided into four groups. The mice were administered with RSG or LPS as described in the Materials and Methods section. Colocalization between PPARγ and NF‐κB p65 was measured in the mouse lungs through IF. Representative pictures of colocalization were shown. Data were expressed as mean ± S.E.M. (*N* = 3). ^∗∗^ *p* < 0.01.

The interaction between PPARγ and NF‐κB p65 was assessed in BEAS‐2B cells with Co‐IP. As shown in Figure 6A, RSG pretreatment alone had no influence on the expression of NF‐κB p65 in immunocomplexes precipitated by anti‐PPARγ, the expression of NF‐κB p65 in immunocomplexes precipitated by anti‐PPARγ was upregulated in LPS‐exposed BEAS‐2B cells. Moreover, RSG plus LPS further increased the expression of NF‐κB p65 in immunocomplexes precipitated by anti‐PPARγ in the nuclei of BEAS‐2B cells (Figure 6A). Concurrently, the colocalization between PPARγ and NF‐κB p65 was evaluated in the mouse lungs through IF. The results indicated that RSG pretreatment promoted PPARγ nuclear translocation in the mouse lungs (Figure 6B). LPS exposure alone elevated NF‐κB p65 nuclear translocation in the lung tissues (Figure 6B). RSG plus LPS co‐culture promoted the colocalization of PPARγ with NF‐κB p65 in the nuclei of mouse lungs (Figure 6B).

## 4. Discussion

It is well known that RSG, as a PPARγ agonist, has many advantages in curing diabetes [14]. Recent studies have shown that RSG treatment can attenuate many inflammatory diseases [15–18]. Our research found a new role of RSG on LPS‐evoked ALI in mice. The results indicated that oral RSG supplementation evidently alleviated LPS‐induced ALI and death in mice. Further analyses hinted that pretreatment with RSG effectively abolished LPS‐activated inflammation, oxidative stress, and ER stress in the mouse lungs. These data indicated that oral RSG supplementation could mitigate LPS‐evoked ALI in mice.

ALI is severe disease characterized by rapid onset and prolonged duration, and poses a vital threat to life. More and more evidence has demonstrated that ALI can be induced by many different factors, such as trauma, bacterial or viral pneumonia, inhaling harmful gas, and so on [1]. LPS, a significant constituent of the Gram‐negative bacterial outer membrane, promotes the production of proinflammatory cytokines [9]. In many studies, LPS has been widely used to estimate the animal model of ALI, which can effectively simulate the pathological features, such as inflammation, oxidative stress, and inflammatory cell infiltration [29]. However, the influence of oral RSG supplementation on LPS‐evoked ALI was not clearly elucidated. Our results showed that RSG pretreatment alleviated LPS‐evoked pulmonary edema and inflammatory cell infiltration and inhibited LPS‐evoked alveolar damage and pathological changes. Moreover, the LPS‐evoked increase in death was significantly attenuated by RSG pretreatment in mice. Therefore, these data suggest that oral RSG supplementation protects from the progression of LPS‐evoked ALI in mice.

A previous investigation has shown that inflammatory signaling pathways are activated in the lungs of LPS‐induced ALI model [13]. NF‐κB, which is one of the most studied transcription factors, modulates a number of cellular events including inflammation, proliferation, and apoptosis. NF‐κB is implicated in several cellular processes, such as infection, stress signaling, and the development of cancer and autoimmune diseases [30]. NF‐κB family is composed of different members; NF‐κB p65 and NF‐κB p50 are the common and important subunits. NF‐κB can regulate the transcription of a large number of genes, consisting of proinflammatory cytokines and chemokines [31]. An earlier report has demonstrated that NF‐κB plays major roles in LPS‐evoked ALI [32]. However, the effect of oral RSG supplementation on the pulmonary NF‐κB signaling pathway during LPS‐evoked ALI is obscure. Our results found that TNF‐α and IL‐1β, two proinflammatory cytokines, were increased in the lungs of LPS‐exposed mice. Moreover, KC, MIP‐2, and MCP‐1, three chemokines, were increased at 12 h after LPS injection. Not only that, the levels of serum TNF‐α and KC increased in LPS‐exposed mice. LPS exposure prominently promoted nuclear translocation of NF‐κB p65 and NF‐κB p50 in the lungs during ALI. Interestingly, oral RSG supplementation significantly mitigated LPS‐evoked the secretion of proinflammatory cytokines and chemokines, and NF‐κB nuclear translocation in the lungs. These results indicate that oral RSG supplementation protects against LPS‐incurred ALI partially through alleviating pulmonary NF‐κB signaling activation.

Mounting evidence has revealed that oxidative stress is implicated in the progression of LPS‐induced ALI [33]. Antioxidants could effectively inhibit LPS‐evoked ALI in the mice [34, 35]. Some studies found that RSG has antioxidant activities [28, 36]. A previous study has demonstrated that RSG pretreatment alleviates burn‐evoked oxidative injury in the remote organs [37]. Nevertheless, whether oral RSG supplementation protected from LPS‐evoked ALI through antioxidant effects is unclear. Our results suggested that pulmonary GSH level was reduced and lipid peroxidation was elevated in the mouse lungs after LPS injection. As expected, oral RSG supplementation attenuated LPS‐evoked GSH depletion and lipid peroxidation in the lungs. Furthermore, NADPH oxidases, which are composed of many membrane‐related subunits, such as P22PHOX, P47PHOX, and P67PHOX, are important enzymatic sources of cellular reactive oxygen species (ROS) [38]. NOX‐2 and NOX‐4, two homologs of NADPH oxidases, are substantially upregulated during LPS‐induced ALI [39, 40]. Besides, HO‐1 and SOD, which are important antioxidant enzymes, can protect against LPS‐incurred ALI [41, 42]. The current investigation indicated that oral RSG supplementation dramatically inhibited the LPS‐evoked upregulation of P47phox mRNA and the expression of NOX‐2 and NOX‐4 in the lungs. Moreover, LPS‐evoked downregulation of Sod‐1 and Sod‐3 mRNAs and upregulation of HO‐1 were effectively restored by RSG pretreatment. Taken together, these results hint that oral RSG supplementation mitigates LPS‐evoked ALI partially through restraining pulmonary oxidative stress. A reduction in pulmonary NADPH oxidase level and an increase in antioxidant enzymes may exert important roles in RSG‐mediated alleviation of oxidative stress during LPS‐evoked ALI.

ER is an important site for protein synthesis, folding, and structural maturation in eukaryotic cells. The aggregation of misfolded and unfolded proteins in the ER incurs an imbalance in homeostasis, and subsequently induces the unfolded protein response (UPR), which is also called ER stress [43, 44]. ER stress is provoked and mediated by three ER transmembrane sensors including inositol‐requiring enzyme 1α (IRE1α), pancreatic endoplasmic reticulum kinase (PERK), and activating transcription factor 6 (ATF6) [45]. When ER stress occurs, the ER molecular chaperone GRP78 dissociates from the three sensors, thus activating three signal pathways and finally incurring the UPR [46]. A previous study demonstrated that ER stress is involved in LPS‐induced ALI [47]. In vivo experiments showed that LPS initiates ER stress in BEAS‐2B cells [48]. In the current study, LPS exposure markedly evoked abnormal expression of ER stress‐related proteins. The levels of GRP78 expression, IRE1α and EIF2α phosphorylation, were raised in the lungs of LPS‐exposed mice. Surprisingly, pretreatment with RSG significantly alleviated LPS‐evoked pulmonary ER stress during ALI. These data indicate that oral RSG supplementation relieves LPS‐initiated ALI partially through downregulating ER stress.

These results hint that oral RSG supplementation protects against LPS‐evoked ALI, partially through inhibiting the inflammatory response, oxidative stress, and ER stress, but the specific mechanism is still not clear. Increasing evidence has suggested that PPARγ inhibits NF‐κB signaling in different cell types [25, 49]. A report from our team has demonstrated that RSG pretreatment alleviates LPS‐evoked inflammatory response and fetal growth restriction by inhibiting the physical interaction between PPARγ and NF‐κB p65 in the cytoplasm and nucleus of placental trophoblasts [25]. Therefore, we examined whether pretreatment with RSG alleviated LPS‐initiated ALI via inhibiting the interaction between PPARγ and NF‐κB p65 in bronchial epithelial cells. Indeed, although RSG pretreatment and LPS exposure had no effect on Pparγ mRNA, RSG pretreatment evidently upregulated PPARγ nuclear translocation in the lungs. Moreover, RSG plus LPS promoted the interaction of NF‐κB p65 with PPARγ in the nuclei of BEAS‐2B cells. Furthermore, RSG plus LPS facilitated the colocalization between NF‐κB p65 and PPARγ in the nuclei of mouse lungs. Therefore, these results indicated that RSG pretreatment mitigated LPS‐evoked pulmonary inflammation in an NF‐κB p65‐dependent manner. In addition, NF‐κB signaling inhibition can alleviate oxidative stress and ER stress [50, 51]. Therefore, it is reasonable to presume that the inhibitory effect of RSG pretreatment on LPS‐induced cellular stress is associated with the interaction between NF‐κB p65 and PPARγ. So, we speculate that RSG pretreatment alleviates LPS‐evoked ALI through inhibiting NF‐κB p65 signaling, oxidative stress, and ER stress in an NF‐κB p65‐dependent manner.

## 5. Conclusion

The current study mainly explored the influence of oral RSG supplementation on LPS‐evoked ALI and the potential mechanisms. Our results showed that oral RSG supplementation alleviated LPS‐evoked ALI and death in mice. In addition, we found that oral RSG supplementation mitigated LPS‐evoked pulmonary inflammation via repressing NF‐κB signaling. Moreover, we found that oral RSG supplementation attenuated LPS‐evoked pulmonary oxidative stress through regulating NAPDH oxidases and antioxidant enzymes. Besides, LPS‐evoked ER stress was markedly inhibited by oral RSG supplementation. Mechanistically, RSG supplementation elevated PPARγ nuclear translocation in the mouse lungs. RSG pretreatment inhibited LPS‐induced NF‐κB p65 activation via promoting the physical interaction between NF‐κB p65 and PPARγ in the mouse lungs. This study provides additional evidence that RSG supplementation protects against LPS‐evoked ALI partially through enhancing PPARγ activity and inhibiting the cellular stress response in the lungs.

## Author Contributions

Participated in research design: Lin Fu, Ling Zheng, Jun Fei, and Jun‐Ping Wei.

Conducted experiments: Jun‐Ping Wei, Wei Cao, Zhen Liu, Zhao‐Fei Liu, Zhen‐Zhen Cao, Xiu Lu, Jia‐Le Li, Min‐Min Tang, and Dong‐Xu Hua

Contributed to reagents/materials/analysis tools: Lin Fu and Ling Zheng.

Performed data analysis and wrote or contributed to the writing of the manuscript: Jun‐Ping Wei and Lin Fu.

## Funding

This study was supported by National Natural Science Foundation of China (82100078), University Natural Science Research Project of Anhui Province (2025AHGXZK31456, 2023AH030117), Anhui Provincial Key Research and Development Project (2022i01020003), Research Funds of Center for Big Data and Population Health of IHM (JKS2023010), and Health Research Project of Fuyang City (FY2023‐013, FY2024‐020).

## Disclosure

All authors read and approved the final manuscript.

## Ethics Statement

All animal experiments were permitted and approved by the Animal Ethics Committee of Anhui Medical University (LLSC20210822).

## Consent

The authors have nothing to report.

## Conflicts of Interest

The authors declare no conflicts of interest.

## Data Availability

The datasets used and/or analyzed during the current study are available from the corresponding author upon reasonable request.
